# Effects of supplementation of vitamin B-complex on performance of beef calves during a 42-day preconditioning program

**DOI:** 10.1093/tas/txag084

**Published:** 2026-06-19

**Authors:** Erica F de Oliveira, Aline C R dos Santos, Ana L P Ramalho, Carlos E M dos Santos, Matheus F L Ferreira, Jeff Heldt, Chance Farmer, Juliana Ranches

**Affiliations:** Eastern Oregon Agricultural Research Center, Oregon State University, Burns, OR, 97720, United States; Eastern Oregon Agricultural Research Center, Oregon State University, Burns, OR, 97720, United States; Eastern Oregon Agricultural Research Center, Oregon State University, Burns, OR, 97720, United States; Eastern Oregon Agricultural Research Center, Oregon State University, Burns, OR, 97720, United States; Hill Farm Research Station, Louisiana State University, Homer, LA, 71040, United States; Selko USA, Indianapolis, IN, 46231, United States; Selko USA, Indianapolis, IN, 46231, United States; Eastern Oregon Agricultural Research Center, Oregon State University, Burns, OR, 97720, United States; Selko USA, Indianapolis, IN, 46231, United States

**Keywords:** cortisol, feed intake, preconditioning, vitamin B-complex, weaned beef calves

## Abstract

This study evaluated the effects of supplementation levels of a blend of vitamin B complex offered to weaned beef calves in a 42-day preconditioning program. Sixty-three Angus × Hereford calves were stratified by body weight (BW; 252 ± 3.7 kg) after weaning (d0) and allocated into 21 pens (3 calves/pen). Pens were randomly assigned to one of three treatments: (1) Control, (2) VitB1g, or (3) VitB2g. Calves assigned to vitamin B supplementation received vitamin B complex (1 vs. 2 g/calf daily; Vivalto, Selko USA; containing pantothenic acid [B5], pyridoxine [B6], folic acid [B9], biotin [B7], cyanocobalamin [B12]) mixed with 1.3 kg of dried distillers’ grains (DDG), while calves in the Control group received only DDG. All calves had free-choice access to chopped alfalfa-grass hay and limited whole corn. Body weights and blood samples were collected from all calves on days 0, 1, 3, 7, 14, 21, and 42 relative to weaning to evaluate plasma cortisol and acute-phase proteins. Vitamin B7 concentration was measured on days 0, 14 and 42. Pen was the experimental unit, and data were analyzed as repeated measures using the MIXED procedure in SAS. No effects of treatment, day, and the interaction (*P* ≥ 0.23) were observed for any of the growth performance variables analyzed. At weaning, all calves had similar (*P* ≥ 0.91) plasma vitamin B7 concentrations, and with two weeks of supplementation, on d14, calves assigned to VitB2g treatment had greater (*P* ≤ 0.005) vitamin B7 concentration than calves assigned to VitB1g and Control treatments, which had similar vitamin B7 concentrations (*P* = 0.79). At the end of the preconditioning phase, on d42, calves assigned to VitB2g had greater (*P* ≤ 0.02) vitamin B7 concentrations than calves assigned to VitB1g and Control treatments, which continued to remain similar (*P* = 0.17). A tendency for a treatment × day (*P* = 0.08) for cortisol concentration was observed. Cortisol concentrations were similar at weaning (*P* ≥ 0.89). Interestingly, at the end of the supplementation, calves supplemented with vitamin B complex, regardless of vitamin level supplementation, had lower cortisol concentration (*P* ≤ 0.05) than calves assigned to the Control treatment. Collectively, these findings suggest that vitamin B complex supplementation during preconditioning enhances circulating vitamin B7 concentration, especially at 2 g/d, and may attenuate physiological stress responses in weaned beef calves.

## Teaser

- Strategies such as preconditioning to reduce stress and improve performance after weaning are warranted.
- Vitamin B-complex supplementation for beef calves during a 42-day preconditioning program increased vitamin B7 concentration in plasma and ameliorated cortisol concentration at the end of the preconditioning program.
- Vitamin B-complex supplementation for beef calves during a 42-day preconditioning program did not improve calf growth.

## Introduction

In beef production, cattle encounter numerous stressful events throughout their lives, such as castration, weaning, transportation, commingling, and temporary restriction of feed and water access (Duff and Galyean 2007). When these stressful conditions coincide with nutritional transitions or suboptimal feeding strategies, they can alter both nutrient demands and digestive efficiency, ultimately decreasing voluntary feed consumption and compromising growth rates (Carroll and Forsberg 2007; Gouvêa et al. 2022). Numerous strategies have been proposed to minimize stress post-weaning, with preconditioning being one of the most widely adopted (Cooke 2017).

Preconditioning refers to targeted management practices implemented in recently weaned calves before they transition to feedlots or market channels. Studies have shown that preconditioning effectively minimizes stress-related challenges while promoting improved health status, welfare, and productivity through reduced morbidity and mortality (Cole 1985; Peterson et al. 1989; Roeber et al. 2001; Step et al. 2008; Mijar et al. 2023). These programs typically incorporate strategic interventions including anthelmintic administration, immunization protocols, and mineral-vitamin supplementation (Peterson et al. 1989).

Vitamins serve critical functions in fundamental physiological processes such as energy metabolism, protein biosynthesis, immune system regulation, reproductive performance, growth, tissue maintenance, and milk production in livestock species (Zinn et al. 1987). These organic micronutrients, though needed in minute quantities, are indispensable for normal physiological function and development in living organisms (Kruse and McCollum 1929). When vitamin intake is insufficient, consequences may include compromised growth rates, diminished productive performance, elevated disease susceptibility, and in cases of severe deficiency, mortality (Devasena et al. 2007). More specifically, vitamins of the B-complex function as essential coenzymes in enzymatic reactions that regulate carbohydrate, lipid, and protein metabolism (McDowell 2000). With the exception of cyanocobalamin (B12) these water-soluble compounds have limited storage in animal tissues, necessitating consistent dietary intake, and in fact, young calves require exogenous vitamin sources as their developing rumen lacks sufficient microbial capacity for vitamin synthesis (NASEM 2016).

The role of B-complex vitamins remains poorly understood, as ruminants were long assumed to meet their B-vitamin requirements through ruminal microbial synthesis. However, B-vitamin synthesis of young calves may be insufficient under conditions of stress, and suboptimal nutrition, such as during weaning and transition from grazing to feeding, where calves might take time adapting to new feeding systems, potentially creating a need for exogenous B-vitamin supplementation (NASEM 2016; Girard and Duplessis 2022).

While preconditioning programs commonly incorporate strategic interventions to enhance calf immunity and growth performance, limited research has addressed B-complex vitamin supplementation during this period. Nevertheless, providing supplemental vitamins during this period may offer advantages for these newly weaned calves, often under stress. Therefore, we hypothesized that B-complex vitamin supplementation for growing calves, specifically during preconditioning, would enhance energy metabolism, thereby increasing feed intake and improving growth performance, while influencing stress markers and other metabolites. Thus, the objective of this study was to evaluate the effects of different levels of B-complex vitamin supplementation on post-weaning growth performance and stress markers of beef calves during a 42-day preconditioning phase.

## Materials and methods

Animal care and handling procedures used in this study were approved by the Institutional Animal Care and Use Committee of Oregon State University (IACUC-2023-0403). The study was conducted during fall 2023 at the Eastern Oregon Agricultural Research Center, Oregon State University (EOARC; Burns, OR; 43°51′86″N, 119°02′15″W).

### Experimental design and calf management

Sixty-three crossbred calves (Hereford × Angus) were selected from EOARC herd to be enrolled in the study. Prior to the initiation of the study, calves were maintained with their dams on 6500-ha semi-arid rangeland pasture at the Northern Great Basin Experimental Range (NGBER, Riley, OR; 43°29ʹ37″N, 119°42ʹ30″W) until weaning (d0). At weaning, calves were weighed (252 ± 3.63 kg BW, 203 ± 16 d of age) and vaccinated against 7-way clostridium, infectious bovine rhinotracheitis (IBR) virus, bovine viral diarrhea (BVD) Type 1 virus, parainfluenza 3 (PI3) virus, bovine respiratory syncytial virus (BRSV) and *Histophilus somni*, with a booster 27 days after weaning. Immediately after weaning and processing, calves were transported 76 km by livestock trailer to the EOARC.

Upon arrival at EOARC, calves were housed as a single group with free-choice access to water and chopped alfalfa-grass hay (CP 15.3% and TDN% 58.8%, approximately 10 cm in length).

On d1, calves were handled at the working facility, weighed, and assigned to one of twenty-one pens (3 calves/pen; 7 pens/treatment). Pens had natural soil flooring (≥7 × 20 m) and concrete feed bunks (7 m linear), equipped with an automatic water tank. The pens were then randomly assigned to one of three treatments: (1) Control, (2) VitB1g, or (3) VitB2g. Calves assigned to the Control treatment served as a negative control and received no vitamin supplementation and only dry distillers’ grains (DDG; 1.3 kg/pen daily). Calves assigned to VitB1g received 1.0 g of vitamin B-complex daily, providing: 36 mg d-pantothenic acid (B5), 22.6 mg pyridoxine (B6), 3.6 mg folic acid (B9), 2.9 mg d-biotin (B7), and 0.4 mg cobalamin (B12). Calves assigned to the VitB2g treatment received 2.0 g of vitamin B-complex daily, providing: 72 mg B5, 45.2 mg B6, 7.2 mg B9, 5.8 mg B7, and 0.8 mg B12. These supplementation values were based on previous feedlot research using the same vitamin B supplementation (Ribeiro et al. 2023).

The B-complex vitamin supplemented (Vivalto, Selko USA, Indianapolis, IN) was fat-encapsulated to avoid ruminal degradation, allowing a 95% bypass rate (as indicated by the manufacturer). The vitamin B supplement was weighed daily and mixed daily into 1.3 kg of DDG and offered at the pen level, starting on d1 and continuing through the end of the preconditioning phase (d42).

### Data and sample collections

Calves were weighed at weaning (d0) and on 1, 3, 7, 14, 21, 41 and 42 of the preconditioning phase. To account for the potential influence of shrink-induced stress on blood parameters, full BW of calves was collected in two consecutive days and used as weaning weight (d0 and 1) and final weight (d41 and 42; Marques et al. 2012). Weight measurements were obtained using a Tru-Test livestock electronic scale (Datamars SA, Auckland, New Zealand). Calf ADG was calculated by subtracting the current BW from the previous BW, then dividing the result by the number of days between weights.

Blood samples were collected via jugular venipuncture on d0, 1, 3, 7, 14, 21, and 42 to measure blood metabolites. Plasma and serum were obtained using commercial heparinized vacuum tubes and tubes without additives, respectively (BD Vacutainer, 10 mL; Becton, Dickinson and Company, Franklin Lakes, NJ). Immediately after collection, samples were placed on ice and transported to the laboratory for processing. Samples were centrifuged at 2,500 x g for 30 minutes at 4°C. The resulting plasma and serum were initially stored at −20°C and subsequently transferred to −80°C for long-term storage until analysis.

For the duration of the study, pen feed intake was adjusted daily to allow minimal refusal without restricting dry matter intake (DMI). The DMI was calculated on a dry matter basis by subtracting feed refusals from feed offered. Calves were fed Alfalfa hay (*Medicago sativa*) ad libitum and limited whole corn daily (approximately 2.7 kg/pen by the end of the preconditioning). Corn inclusion gradually increased, with dietary adjustments on d 7 and d 21, to minimize the risk of ruminal disturbances. Corn and hay were offered separately to allow quantification of intake for each feed ingredient. Corn was offered in a smaller trough placed inside the pen feed bunk. Alfalfa hay samples were collected weekly throughout the entire preconditioning program and composited. Hay samples were dried in a forced-air oven at 55°C for 72 h, then ground to 1- and 2-mm particle sizes using a Wiley mill (Model 3; Arthur H. Thomas Co., Philadelphia, PA). Ground samples were submitted to a commercial laboratory (Dairy One Forage Laboratory, Ithaca, NY) for nutrient analysis. Crude protein (CP) concentrations were determined using wet chemistry procedures (AOAC, 2006; method 984.13). Neutral detergent fiber (NDF) was analyzed according to Van Soest et al. (1991), modified for use with an Ankom 200 fiber analyzer (Ankom Technology Corp., Macedon, NY). Total digestible nutrients (TDN) were estimated using the equations described by Weiss et al. (1992). Alfalfa-grass hay provided 15.3% of crude protein (CP) and 58.8% total digestible nutrients (TDN); 1.10%, 0.20%, 0.20%, 1.60%, 0.20% of DM for Ca, P, Mg, K, and Na, respectively; and 805.30, 24.80, 7.30, 137.80, 2.90, 1.20 ppm Fe, Zn, Cu, Mn, Mo, and Co respectively.

### Laboratory analyses

Serum concentrations of ceruloplasmin, plasma concentrations of cortisol, and haptoglobin were evaluated on d0, 1, 3, 7, 14, 21, and 42 as indicators of acute phase proteins and stress, as previously conducted (Ferreira et al. 2024). Vitamin B7 concentration in plasma was measured on d0, 14, and 42.

Serum ceruloplasmin was measured in duplicate using a colorimetric assay based on the protocol of (Demetriou et al. 1974), with values reported in mg/dL. Intra-assay and inter-assay CV were 11.14% and 4.41%, respectively.

Plasma cortisol was measured by chemiluminescent immunoassay (Immulite 1000; Siemens Medical Solutions Diagnostics, Los Angeles, CA) and reported in ng/mL. The intra-assay CV was 5.59%.

Plasma haptoglobin was measured using a colorimetric method based on haptoglobin-hemoglobin binding and peroxidase activity (Makimura and Suzuki 1982). Duplicate samples were analyzed, and absorbance readings at 450 nm (VersaMax Tunable EXT) were recorded as relative units. To validate the assay and establish quality control parameters, bovine haptoglobin standards were quantified using a commercial ELISA kit (Life Diagnostics, Inc., West Chester, PA). Control samples ranged from 0.28 µg/mL (low) to 1.16 µg/mL (high), with intra- and inter-assay coefficients of variation of 2.02% and 1.69%, respectively. Arbitrary units from the colorimetric assay were converted to µg/mL using an ELISA standard curve (Cooke and Arthington 2013). The minimum detection limit was 0.03 µg/mL. For the colorimetric assay, intra- and inter-assay CV were 3.1% and 9.3%, respectively.

Vitamin B7 plasma concentrations, as evaluated by others (Duplessis et al. 2024), were measured on d0, 14, and 42 using a commercial competitive ELISA kit (Biomatik, Cat. No. EKU11525, Kitchener, ON, Canada). According to the manufacturer, the assay exhibits universal species reactivity and is suitable for serum and plasma samples from multiple species. In this competitive assay, vitamin B7 present in the sample competes with plate-bound antigen for binding to a horseradish peroxidase-conjugated detection reagent. Following substrate addition, color development was measured at 450 nm using a VersaMax Tunable Microplate Reader (Molecular Devices, San Jose, CA). Plasma samples were diluted 1:10 prior to analysis. The inter-assay coefficient of variation was 11%.

### Statistical analysis

Growth performance variables were analyzed using pen as the experimental unit, with pen nested within treatment included as a random effect. Variables collected repeatedly over time were analyzed using the MIXED procedure of SAS (SAS Inst. Inc., Cary, NC), including the fixed effects of treatment, day, and treatment × day. Calf was specified as the subject for repeated measures, and several covariance structures were evaluated. Compound symmetry was selected for the final model based on the lowest Akaike Information Criterion (AIC). Single-measurement variables, such as weaning and final BW, and ADG, were analyzed using a model containing treatment as a fixed effect and pen nested within treatment as a random effect. Least squares means were separated using the PDIFF option. Significance was declared at *p* ≤ 0.05 and tendencies at 0.05 < *p* ≤ 0.10.

## Results

No effects of treatment, day, or treatment × day were observed for weaning BW (252, 249, and 252 ± 3.64 kg, respectively for Control, VitB1g, and VitB2g; *p* = 0.82), final BW (283, 283, and 284 ± 3.79 kg; *p* = 0.98), or overall ADG (0.735, 0.795, and 0.747 ± 0.039 kg/d; *p* = 0.53; Table 1).

**Table 1:** Effect of vitamin B-complex supplementation on body weight and average daily gain of calves during preconditioning.

		Treatments^a^		SEM	*P*-value
Item	Control	VitB1g	VitB2g		
***Weaning BW^2^, kg***	252	249	252	3.636	0.82
***Final BW^2^, kg***	283	283	284	3.789	0.98
***ADG^3^, d0 to 14 kg/d***	1.87	2.23	2.11	0.150	0.26
***ADG^3^, d0 to 21 kg/d***	1.21	1.44	1.37	0.093	0.23
***ADG^3^, d14 to 21 kg/d***	0.899	1.01	0.960	0.379	0.97
***ADG^3^, d21 to 42 kg/d***	0.299	0.210	0.185	0.094	0.66
***Overall ADG^3^, kg/d***	0.735	0.795	0.750	0.039	0.53

a Control: calves received 1.3 kg of dried distillers grains (DDG) per day; VitB1g or VitB2g: calves received vitamin B-complex (1 or 2 g/calf daily; Vivalto, Selko USA) mixed with 1.3 kg of DDG per day; *n* = 21 pens (7 pens/treatment; 3 calves/pen. Assigning weaning as d0, vitamin B-complex supplementation was provided from d0 to 42 during preconditioning.

b BW, body weight. Initial BW was calculated as the average of BW measured on d0 and d1, and final BW was calculated as the average of BW measured on d41 and d42.

c ADG, average daily gain, calculated as (final BW − initial BW)/42 d.

No treatment or treatment × day were observed for total feed intake (16.7, 16.2, and 16.7 ± 0.262 kg/pen/d, respectively for Control, VitB1g, and VitB2g; *p* = 0.58), hay intake (14.5, 14.1, and 14.5 ± 0.264 kg/pen/d; *p* = 0.63), or corn intake (2.14, 2.12, and 2.13 ± 0.007 kg/pen/d; *p* = 0.51). A day effect was observed for all intake variables (*p* < 0.001), with feed intake increasing proportionally to BW as calves progressed through the preconditioning program (Table 2).

**Table 2:** Effect of vitamin B-complex supplementation on total dry matter intake (DMI), hay intake, and corn intake per pen during preconditioning.

	Treatments^a^				*P*-value		
Item	Control	VitB1g	VitB2g	SEM	Treatment	Day	Treatment × Day
***Total DMI^b^, kg/pen/d***	16.7	16.2	16.7	0.262	0.33	<.0001	0.58
***Hay DMI^b^, kg/pen/d***	14.5	14.1	14.5	0.264	0.35	<.0001	0.52
***Corn DMI^b^, kg/pen/d***	2.14	2.12	2.13	0.007	0.49	<.0001	0.63

a Control: calves received 1.3 kg of dried distillers’ grains (DDG) per day; VitB1g or VitB2g: calves received vitamin B-complex (1 or 2 g/calf daily; Vivalto, Selko USA) mixed with 1.3 kg of DDG per day; *n* = 21 pens (7 pens/treatment; 3 calves/pen. Assigning weaning as d0, vitamin B-complex supplementation was provided from d0 to 42 during preconditioning. Values are reported as treatment means.

b DMI, dry matter intake.

For the acute phase proteins ceruloplasmin and haptoglobin, no treatment effects (*p* ≥ 0.42) or treatment × day were observed (*p* ≥ 0.57). A day effect was observed for both ceruloplasmin and haptoglobin (*p* < 0.001; Table 3). Plasma ceruloplasmin concentrations increased (*p* < 0.001) from d1 (25.1 ± 0.82 mg/mL) to d3 (28.9 ± 0.82 mg/mL), peaked at d7 (33.0 ± 0.82 mg/mL; *p* < 0.001), and subsequently declined (*p* < 0.001), returning to baseline levels by d42 (26.3 ± 0.82 mg/mL). Similarly, plasma haptoglobin concentrations increased (*p* < 0.001) from d0 (0.175 ± 0.05 µg/mL) to d1 (0.744 ± 0.05 µg/mL), peaked at d3 (2.042 ± 0.05 µg/mL; *p* < 0.001), and subsequently declined to d7 (0.293 ± 0.05 µg/mL; *p* < 0.001), remaining stable thereafter and returning to baseline levels by d42 (0.231 ± 0.05 µg/mL; *p* ≥ 0.37).

**Table 3:** Effect of vitamin B-complex supplementation on acute-phase proteins and cortisol of calves during preconditioning.

		Preconditioning days								*P*-value		
Item	Treatment^1^	Day 0	Day 1	Day 3	Day 7	Day 14	Day 21	Day 42	SEM	Treatment	Day	Treatment × Day
***Ceruloplasmin, mg/mL***		24.5^d^	25.1^d, c^	28.9^b^	33.0^a^	28.5^b^	26.3^c^	26.3^c^	0.82	0.66	<.0001	0.59
***Haptoglobin, µg/mL***		0.175^d^	0.744^b^	2.042^a^	0.293^c, d^	0.337^c^	0.328^c^	0.231^c, d^	0.05	0.41	<.0001	0.56
***Cortisol, ng/mL***	Control	2.02	2.26	1.69^x^	1.77	1.88	1.62	2.46 ^x^	0.173	0.72	<.0001	0.08
	VitB1g	2.05	2.45	2.18^y^	1.79	1.70	1.49	2.00 ^y^				
	VitB2g	2.00	2.17	1.77^x, y^	1.64	1.63	1.74	1.90 ^y^				

a–d Within a row, different superscripts show differences between days (*p* ≤ 0.05).

x–y Within a column, different superscripts show differences between treatments (*p* ≤ 0.05).

1 Control: calves received 1.3 kg of dried distillers’ grains (DDG) per day; VitB1g or VitB2g: calves received vitamin B-complex (1 or 2 g/calf daily; Vivalto, Selko USA) mixed with 1.3 kg of DDG per day. Assigning weaning as d0, vitamin B-complex supplementation was provided from d1 to 42 during preconditioning.

For cortisol concentrations, no treatment effects (*p* = 0.72) were observed. However, a day effect was found (*p* < 0.001), with a tendency for a treatment × day (*p* = 0.08, Table 3). Concentrations were similar at weaning (d0; *p* ≥ 0.89; 2.02 ± 0.173 ng/mL). On d3, cortisol concentrations were greater for calves assigned to VitB1g treatment (2.18 ± 0.172 ng/mL, *p* = 0.04) compared to calves assigned to Control treatment (1.69 ± 0.172 ng/mL), with calves assigned to VitB2g treatment being intermediate (1.77 ± 0.172 ng/mL, *p* = 0.11). At the end of the supplementation period (d42), cortisol concentrations were greater for calves assigned to Control treatment compared to calves assigned to vitamin B supplementation, regardless of the level of supplementation (2.46, 2.00, and 1.90 ± 0.172 ng/mL, respectively, for Control, VitB1g, and VitB2g; *p* = 0.05).

**Figure 1:**
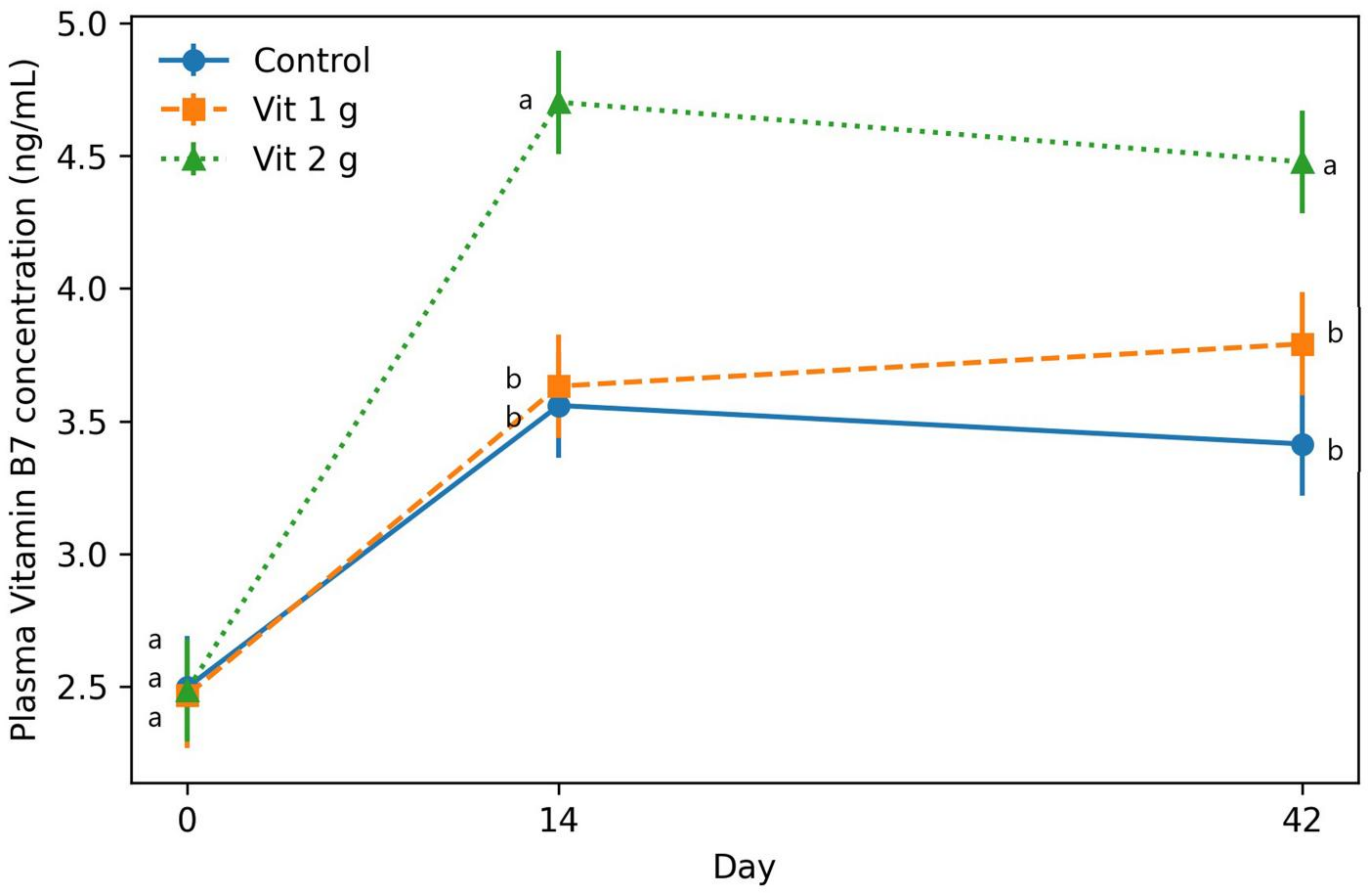
Plasma vitamin B7 concentrations of beef calves supplemented with vitamin B-complex during a 42-day preconditioning program. Treatment (*p* = 0.0057), day (*p* < 0.001), and treatment × day (*p* = 0.0008) effects were observed. At d0, all treatments were similar (*p* = 0.91). At d14 and d42, VitB2g had greater vitamin B7 concentrations than VitB1g and control (*p* ≤ 0.02), while VitB1g and control did not differ at either timepoint (*p* ≥ 0.17). values are expressed as LSMeans ± SEM (*n* = 21). control = no vitamin supplementation (*n* = 21); VitB1g = 1 g of rumen-protected B-complex vitamins (*n* = 21); VitB2g = 2 g of rumen-protected B-complex vitamins (*n* = 21). different superscript letters (a, b) within a day indicate significant differences among treatments (*p* < 0.05).

Regarding plasma vitamin B7 concentration, there was a treatment effect (*p* = 0.0057), a day effect (*p* < 0.001) and a treatment × day (*p* = 0.0008; Fig. 1). At weaning (d0), all calves had similar plasma vitamin B7 concentrations (2.50 ± 0.198 ng/mL; *p* = 0.91). At d14, calves assigned to VitB2g had greater vitamin B7 concentrations (4.70 ± 0.194 ng/mL) than calves assigned to VitB1g (3.60 ± 0.194 ng/mL; *p* = 0.0003) and Control treatments (3.50 ± 0.194 ng/mL; *p* = 0.0001); while calves assigned to VitB1g and Control did not differ (*p* = 0.80). At the end of the preconditioning program, on d42, calves assigned to VitB2g had greater vitamin B7 concentrations (4.50 ± 0.194 ng/mL) than calves assigned to VitB1g (3.80 ± 0.194 ng/mL; *p* = 0.01) and Control (3.41 ± 0.194 ng/mL; *p* = 0.0003), while plasma vitamin B7 of calves assigned to VitB1g and Control treatments were similar (*p* = 0.17).

## Discussion

Plasma vitamin B7 concentrations were measured to assess the bioavailability of the supplemented B-complex vitamins and ensure supplement consumption. Vitamin supplementation at 2 g of B-complex vitamins (VitB2g) positively influenced plasma B7 concentration compared to supplementation of 1 g (VitB1g) and Control. However, supplementation at 1 g did not consistently differ from Control, suggesting that supplementation at 1 g might be insufficient in this system, or may have been utilized to support metabolic demands rather than accumulating in circulation. The increased plasma B7 concentrations in calves assigned to VitB2g treatment confirm that the fat-encapsulated formulation successfully bypassed ruminal degradation and was absorbed in the small intestine, which is consistent with Deters et al. (2021), who demonstrated that rumen-protected B-complex formulations achieve greater intestinal absorption. As proposed by Santschi et al. (2005), non-protected B-vitamin supplements are more susceptible to ruminal degradation and microbial utilization, highlighting the importance of rumen protection for effective supplementation.

Supplementation with B7 has been shown to substantially elevate circulating concentrations, with increases of approximately three- to fourfold reported when cows received 20 mg/d (Zimmerly and Weiss 2001; Higuchi et al. 2004), though the biological implications of such elevated concentrations for metabolic function remain incompletely understood. As a cofactor for carboxylase enzymes, vitamin B7 is essential for energy metabolism, participating in gluconeogenesis, fatty acid synthesis, and amino acid metabolism (Rodriguez-Melendez and Zempleni 2003).

While mature ruminants typically rely on microbial synthesis of B-vitamins (Bechdel et al. 1928), production can be influenced by several factors, including dietary composition, stress, and ruminal development (Hayes et al. 1966). It has been proposed that enhanced B7 availability may support gluconeogenic capacity through increased carboxylase enzyme activity, though direct effects on circulating glucose have not been consistently demonstrated, potentially due to tissue uptake and utilization (Zimmerly and Weiss 2001). Recently weaned calves may have reduced capacity for adequate microbial B-vitamin synthesis, due to reduced feed intake, which could make them responsive to exogenous supplementation during periods of stress (Savage and McCay 1942; Dubeski et al. 1996).

The transient cortisol increases on d3 coincided with increases in acute phase proteins haptoglobin (d1 to d3) and ceruloplasmin (d3 to d7), indicating a coordinated stress and inflammatory response in the early postweaning period. Nonetheless, vitamin B-complex supplementation reduced cortisol concentrations by d42, suggesting an impact on stress physiology, as calves assigned to both vitamin B-complex levels had lower cortisol than calves assigned to the Control treatment. Cortisol is a well-established stress biomarker in cattle, reflecting activation of the hypothalamic-pituitary-adrenal (HPA) axis during weaning, a multifactorial stressor involving maternal separation, transportation, social regrouping, and dietary transitions (Carroll and Forsberg 2007). Circulating cortisol is part of the homeostatic response, mobilizing nutrients from tissues to support metabolic adaptation during acute stress. However, chronic cortisol elevation suppresses immune function and reduces animal performance (Hickey et al. 2003). Although the cortisol reduction did not translate into improved growth performance, this metabolic response may have practical implications for calf welfare and subsequent adaptation. Elevated cortisol during the post-weaning period has been associated with immunosuppression, increased disease susceptibility, and reduced vaccine efficacy (Arthington et al. 2003). The lower cortisol concentrations by d42 in vitamin B-supplemented calves may indicate improved stress adaptation that could benefit calves during subsequent challenges such as feedlot entry, where the cumulative effects of chronic stress exposure are known to negatively impact health and performance (Duff and Galyean 2007). This suggests that B-complex vitamin supplementation may serve a protective role in preparing calves for the stressors associated with feedlot adaptation, even when growth benefits are not immediately apparent during preconditioning.

The mechanism by which B-vitamin supplementation reduces cortisol is not entirely clear. Vitamin B5, one of the B-vitamins in the supplement, plays an essential role in corticosteroid hormone synthesis as the precursor of coenzyme A (Tahiliani and Beinlich 1991). Vitamin B6, also present in the supplement, serves as a cofactor for glutamate decarboxylase, the enzyme responsible for converting glutamate to GABA, a primary inhibitory neurotransmitter that modulates HPA axis activity and stress responses (McCarty 2000). Additionally, B6 has been demonstrated to interact with glucocorticoid receptors, potentially down-regulating their activity in rodent models, and has been shown to modulate neurotransmitter synthesis more broadly (Dakshinamurti et al. 1990; McCarty 2000). The combination of these roles in corticosteroid synthesis, HPA axis modulation, and energy metabolism may collectively enhance cattle capacity to adapt to metabolic stress during the post-weaning period, though the specific mechanisms require further investigation.

Despite increases in plasma B7 and reductions in cortisol, vitamin B-complex supplementation did not affect BW, ADG, or total feed intake. B-vitamin complex supplementation of beef cattle has yielded inconsistent results across studies, with outcomes varying according to product composition, dose, stress level, and production stage. Ribeiro et al. (2023), utilizing the same rumen-protected B-complex blend and doses as the present study, supplemented 246 finishing steers in a feedlot setting for 126 days. During the first 28 days, steers supplemented at 1 g/head daily had greater ADG and improved feed efficiency compared to steers assigned to Control and supplemented at 2 g/head daily, however, this response did not last until the end of the study. These findings suggest that the performance response to B-vitamin supplementation may be transient and context-dependent, varying according to diet composition, production stage, and level of metabolic demand.

Using a more similar cattle category as the current study, Zinn et al. (1987) supplemented 144 crossbred feeder calves (116 kg) subjected to long-distance transport stress with two levels of B-vitamin complex, providing 20 mg/d of B1, 40 mg/d of B2, 200 mg/d of B3, 10 mg/d of B9, 2 mg/d of B7, 1000 mg/d of choline, and 0.2 mg/d of B12 at the lower level, and ten times these amounts at the greater level. Contrary to the current study, the authors observed that during the first 28 days, supplemented calves tended to consume less feed, resulting in slightly lower weight gain; however, by day 56, no differences among treatments were observed for BW, DMI, or feed efficiency.

Interestingly, Do et al. supplemented 18 Japanese Black steers from 16 to 28 months of age with rumen-unprotected B9 at either 500 or 1000 mg/day. All groups received 1.5 to 2.6 mg Co/kg DM to prevent vitamin B12 deficiency. Supplementation resulted in greater final BW compared with cattle assigned to the Control treatment. The authors suggested that the positive effects were associated with the role of B9 in regulating growth-related pathways and nutrient metabolism and noted that the unprotected form required substantially greater supplementation rates to compensate for extensive ruminal degradation. In the current study, although B9 was included in the supplement and was rumen protected, supplementation rates were considerably lower (3.6 and 7.2 mg/day for VitB1g and VitB2g, respectively) than those utilized by Do et al. (2024), which may have limited the potential for observing similar performance responses.

In agreement with the current study, Word et al. supplemented 2,091 crossbred yearling steers with 3 mg/kg DM of rumen-protected B9 plus 1.0 mg/kg DM cobalt for 158 days and reported no effects on growth performance. However, carcass-adjusted feed efficiency tended to improve, dressing percentage increased, and liver abscess incidence tended to decrease, suggesting potential improvements in overall cattle health and metabolic efficiency.

The inconsistency in outcomes regardless of vitamin B-complex supplementation has been attributed to factors affecting microbial synthesis and metabolic demands, primarily stress severity and nutritional status (Zinn et al. 1987; Dubeski et al. 1996; Galyean et al. 1999). Most B-vitamin research in beef cattle has focused on feedlot-receiving calves exposed to severe stressors, including long-distance transport, feed restriction, abrupt dietary transitions, and commingling (Cole et al. 1979; 1982; Zinn et al. 1987; Dubeski et al. 1996). Under these conditions, ruminal function may be compromised and microbial synthesis impaired, while metabolic demands and disease pressure, particularly from bovine respiratory disease (BRD) is substantially increased (Duff and Galyean 2007). Dubeski et al. (1996) demonstrated that both feed restriction and viral immune challenge reduced plasma concentrations of B5, B6, and B12 in stressed transported calves, suggesting that severe stress may simultaneously increase physiological requirements for B-vitamins while limiting ruminal microbial synthesis through reduced substrate availability. Some studies that found a lack of performance response from B-vitamin supplementation have reported benefits on reduced morbidity in stressed feeder calves (Cole et al. 1979; 1982; Zinn et al. 1987), suggesting that health benefits, potentially through reduced immunosuppression, may occur independently of performance response.

The present study evaluated preconditioned weaned calves subjected to lower stress intensity. Calves were transported only 76 km, were not commingled with a different herd, and were fed ad libitum with high-quality forage, which likely supported adequate microbial B- vitamin synthesis. Additionally, dams were in adequate body condition at weaning, ensuring sufficient nutrient transfer prior to weaning via milk (Savage and McCay 1942; Kon and Porter 1954). This favorable condition may explain the absence of performance benefits despite metabolic effects on cortisol and circulating B7, suggesting that supplementation may be most beneficial when animals are substantially challenged by metabolic demands and microbial synthesis can be impaired. Future research is warranted to evaluate vitamin B-complex supplementation across the full production cycle under varying stress intensities to fully understand when and how supplementation might be the most effective.

## Conclusion

Vitamin B-complex supplementation during preconditioning increased plasma vitamin B7 concentrations, ameliorated cortisol concentrations by the end of the preconditioning phase, but did not affect acute phase proteins, feed intake, or subsequent calf growth performance.

## List of Abbreviations

ADG: Average daily gain
BRD: bovine respiratory disease
BRSV: bovine respiratory syncytial virus
BVD: bovine viral diarrhea
BW: body weight
CP: crude protein
DDG: dried distillers’ grains
DM: dry matter
DMI: dry matter intake
ELISA: enzyme-linked immunosorbent assay
EOARC: Eastern Oregon Agricultural Research Center
GABA: gamma-aminobutyric acid
HPA: hypothalamic-pituitary-adrenal (axis)
IBR: infectious bovine rhinotracheitis
PI3: parainfluenza-3
SA: serum amyloid A
TDN: total digestible nutrients
